# Non-linear association of fasting C-peptide and uric acid levels with renal dysfunction based on restricted cubic spline in patients with type 2 diabetes: A real-world study

**DOI:** 10.3389/fendo.2023.1157123

**Published:** 2023-03-23

**Authors:** Lu Chen, Yifei Hu, Yongjun Ma, Huabin Wang

**Affiliations:** ^1^ Department of Clinical Laboratory, Affiliated Jinhua Hospital, Zhejiang University School of Medicine, Jinhua, Zhejiang, China; ^2^ Central Laboratory, Affiliated Jinhua Hospital, Zhejiang University School of Medicine, Jinhua, Zhejiang, China

**Keywords:** C-peptide, non-linear association, renal dysfunction, type 2 diabetes, uric acid

## Abstract

**Background:**

Previous studies had showed divergent findings on the associations of C-peptide and/or uric acid (UA) with renal dysfunction odds in patients with type 2 diabetes mellitus (T2DM). We hypothesized that there were non-linear relationships between C-peptide, UA and renal dysfunction odds. This study aimed to further investigate the relationships of different stratification of C-peptide and UA with renal dysfunction in patients with T2DM.

**Method:**

We conducted a cross-sectional real-world observational study of 411 patients with T2DM. The levels of fasting C-peptide, 2h postprandial C-peptide, the ratio of fasting C-peptide to 2h postprandial C-peptide (C0/C2 ratio), UA and other characteristics were recorded. Restricted cubic spline (RCS) curves was performed to evaluated the associations of stratified C-peptide and UA with renal dysfunction odds.

**Results:**

Fasting C-peptide, C0/C2 ratio and UA were independently and significantly associated with renal dysfunction in patients with T2DM as assessed by multivariate analyses (p < 0.05). In especial, non-linear relationships with threshold effects were observed among fasting C-peptide, UA and renal dysfunction according to RCS analyses. Compared with patients with 0.28 ≤ fasting C-peptide ≤ 0.56 nmol/L, patients with fasting C-peptide < 0.28 nmol/L (OR = 1.38, p = 0.246) or fasting C-peptide > 0.56 nmol/L (OR = 1.85, p = 0.021) had relatively higher renal dysfunction odds after adjusting for confounding factors. Similarly, compared with patients with 276 ≤ UA ≤ 409 μmol/L, patients with UA < 276 μmol/L (OR = 1.32, p = 0.262) or UA > 409 μmol/L (OR = 6.24, p < 0.001) had relatively higher odds of renal dysfunction.

**Conclusion:**

The renal dysfunction odds in patients with T2DM was non-linearly associated with the levels of serum fasting C-peptide and UA. Fasting C-peptide and UA might have the potential role in odds stratification of renal dysfunction.

## 1 Introduction

In china, the incidence of diabetes mellitus has rapidly risen from 0.67% in 1980 to 11.2% in 2020, and its prevalence is increasing every year (1). Diabetic kidney disease (DKD) occurs in 20% to 40% of patients with diabetes, it has become one of the most common complications of diabetes and the leading cause of end-stage renal disease in most of the world (2, 3). DKD has posed a rapidly growing global health care burden (4, 5). Therefore, in order to explore the better preventive and therapeutic methods, and lifestyle of patients with DKD, many researchers have focused on studying the clinical risk factors for occurrence and progression of DKD in patients with diabetes, such as age, gender, blood pressure, glycemic control, diabetes duration, body mass index (BMI), and dyslipidemia, among others (6–8).

C-peptide consists of 31amino acids, and is formed by the cleavage of pro-insulin (9). Compared with insulin, C-peptide is secreted in equimolar amounts and has a longer half-life, hence, it is recognized as a reliable indicator of the function of pancreatic β-cell (10). Recently, C-peptide is no longer considered an inert molecule, it may have multiple functions, including signaling pathways activation, physiological effects, and protection against complications of diabetes (11). Animal studies have demonstrated that C-peptide can improve functional abnormalities in the glomeruli, reduce the risk of albuminuria and hyper-filtration, and prevent the chronic complications of diabetes (9, 12). However, several small-scale clinical trials and/or animal studies showed the divergent findings in the role of C-peptide administration in the outcomes of patients with DKD (9). Therefore, the association of serum C-peptide with the risk of DKD is complicated, and it needs to be further clarified in patients with diabetes mellitus.

More recently, some studies have identified serum uric acid (UA) as an independent clinical risk factor for chronic kidney disease in patients with diabetes mellitus (13, 14), however, others do not (15, 16). The association between kidney impairment risk and the different stratification of serum UA level in patients with type 2 diabetes mellitus (T2DM) remains unclear. Given the above, we hypothesized that there were non-linear relationships between C-peptide, UA and renal dysfunction odds. Therefore, we conducted a real-world observational study to further explore the relationships of different stratification of C-peptide and UA levels with renal dysfunction in patients with T2DM.

## 2 Materials and methods

### 2.1 Study design and population

This cross-sectional real-world observational study was based on 513 patients with T2DM who visited at the Endocrinology Department of the Affiliated Jinhua Hospital, Zhejiang University School of Medicine, between January 2017 and December 2017 (17, 18). The subjects of this study were randomly recruited based on the following exclusion criteria: (1) patients with age < 18 years old; (2) patients with primary kidney diseases before the diagnosis of T2DM; (3) patients with serious autoimmune diseases, liver diseases and/or infectious diseases. In addition, 102 subjects with lack of the laboratory data of fasting C-peptide, 2h postprandial C-peptide and/or UA were excluded. Finally, 411 participants with an average age of 57.60 ± 13.52 years old were included in this study. Among the subjects cohort, the median diabetic duration was 7 years; 41.12% of them were female, 58.88% were male, and 48.48% had hypertension; the proportion of participants with angiotensin converting enzyme inhibitor (ACEI) or angiotensin receptor blocker (ARB) use was 27.98%. The present study followed the tenets of the Declaration of Helsinki and was approved by the Ethics Committee of Affiliated Jinhua Hospital, Zhejiang University School of Medicine. Ethical approval code: (Research) 2021-Ethical Review-75-01.

### 2.2 Clinical characteristics and definitions

Most clinical information and the laboratory parameters of the subjects were obtained from the electronic medical record system. The clinical characteristics included gender, age, weight, height, diabetic duration, hypertension, diastolic blood pressure (DBP), systolic blood pressure (SBP), and the history of ACEI/ARB use. The laboratory parameters included glycated hemoglobin (HbA1c), serum UA, creatinine, fasting C-peptide, 2h postprandial C-peptide, triglycerides, total cholesterol, high-density lipoprotein cholesterol (HDL-C), low-density lipoprotein cholesterol (LDL-C), alanine aminotransferase, aspartate aminotransferase. Among these parameters, the HbA1c level was tested by BIO-RAD D-100 analyzer, the other indicators were detected using Beckman Coulter automatic immuno analyzer or automatic biochemical Analyzer with its original reagents in the department of clinical laboratory. The first or second urine specimens of all the subjects were collected, the levels of urinary creatinine and albumin were detected using Beckman Coulter biochemical Analyzer and Byron Diagnostics reagents (Byron, Shanghai, China).

Body mass index (BMI) was calculated based on the height and weight of the participants. The ratio of fasting C-peptide to 2h postprandial C-peptide was defined as C0/C2 ratio. The Xiangya equation (19) based on a large Chinese population was used to calculate the estimated glomerular filtration rate (eGFR). eGFR decline was defined as eGFR < 60 ml/min/1.73m^2^. Albuminuria was defined as urinary albumin-to-creatinine ratio (ACR) > 30 mg/g. In the present study, renal dysfunction was defined as albuminuria and/or eGFR decline.

### 2.3 Statistical analysis

All the statistical analyses of the data in this study were performed by using R software (3.6.4 version) and SPSS 26.0 statistical software. The continuous variables with normally distribution were expressed as mean ± standard deviation (SD); the continuous variables with skewed distribution were expressed as median (Q1 - Q3); the categorical variables were presented as the number (percentage, %). Unpaired t-tests, Mann-Whitney tests and Pearson’s chi-squared tests were performed to execute the between-group comparisons, as appropriate. Logistic regression was conducted for evaluating the relationships between different clinical parameters and the renal dysfunction in patients with T2DM. In addition, because the range of fasting C-peptide values was relatively small (0.02 - 1.53 nmol/L), and the range of serum UA values was relatively wide (148 - 834 μmol/L), hence, fasting C-peptide and UA analyses were also presented as Z-scores and quartiles in the logistic regressions. Restricted cubic spline (RCS) curves based on logistic regression were performed to evaluated the associations of C-peptide and UA values with renal dysfunction odds (odds ratio, OR). According to Akaike Information Criterion (AIC), the knot number corresponding to the minimum AIC value was defined for each model, respectively. In this study, the optimal numbers of knots were 3 for the estimated models, respectively, and these 3 knots located at 10th, 50th, and 90th percentile of the variable distribution were analyzed. The Wald Chi-square test was used to assess both “the overall” and the “nonlinearity” hypothesis in the RCS analyses.

The R package called ‘CatPredi’ was used to categorize the continuous predictors (UA and fasting C-peptide) with “Addfor” algorithm (20, 21), and the optimal cut-points were selected out. In this study, according to the RCS analyses, we considered categorizing the UA and fasting C-peptide variables into 3 categories, respectively. Finally, the threshold effects of fasting C-peptide and UA for renal dysfunction odds based on RCS analysis were also evaluated using logistic regression. A two-tailed p-value < 0.05 was considered statistically significant.

## 3 Results

### 3.1 Characteristics of the participants

Overall the 411 participants with T2DM, 179 (43.55%) had albuminuria, 26 (6.33%) had eGFR decline, the proportion of subjects with renal dysfunction was 183(44.53%). The comparisons of the clinical characteristics between the participants with and without renal dysfunction were summarized in **Table 1**. The levels of age, SBP, HbA1c, diabetic duration, fasting C-peptide, C0/C2 ratio, UA, serum and urinary creatinine, ACR, eGFR and the proportion of hypertension presented statistically significant differences between participants with and without renal impairment (p < 0.05). However, statistically significant differences between the two groups were not detected for 2h postprandial C-peptide, BMI, DBP, gender, triglycerides, total cholesterol, HDL-C and LDL-C. Although the proportion of ACEI/ARB use was also higher in subjects with renal dysfunction compared with the other group (35.52% vs 21.93%, p = 0.002), of note, almost all the participants with ACEI/ARB use were the patients with hypertension.

**Table 1:** Clinical characteristics of study patients.

Variables	Patients without renal dysfunction (n = 228)	Patients with renal dysfunction (n = 183)	p-value
Age, years	55.80 ± 12.57	59.83 ± 14.34	0.003
Male, n (%)	137 (60.09%)	105 (57.38%)	0.579
BMI, kg/m2	24.28 ± 3.28	24.51 ± 3.30	0.492
SBP, mmHg	133 ± 19	138 ± 20	0.010
DBP, mmHg	79 ± 11	79 ± 12	0.868
Hypertension, n (%)	88 (38.60%)	110 (60.11%)	< 0.001
ACEI/ARB use, n (%)	50 (21.93%)	65 (35.52%)	0.002
Diabetic duration, years	7.05 ± 5.66	9.55 ± 7.88	< 0.001
HbA1c, %	8.84 ± 2.44	9.36 ± 2.63	0.041
Total Cholesterol, mmol/L	4.53 ± 1.32	4.41 ± 1.21	0.343
Triglyceride, mmol/L	1.54 (1.00 - 2.24)	1.45 (1.03 - 2.29)	0.884
HDL-C, mmol/l	1.19 ± 0.27	1.16 ± 0.32	0.288
LDL-C, mmol/l	2.92 ± 0.80	2.91 ± 0.94	0.944
Alanine aminotransferase, U/L	19.00 (13.85 - 27.65)	19.00 (12.90 - 25.75)	0.590
Aspartate aminotransferase, U/L	20.20 (16.00 - 25.78)	20.00 (15.50 - 25.95)	0.131
Fasting C-peptide, nmol/L	0.44 ± 0.26	0.52 ± 0.35	0.017
2h postprandial C-peptide, nmol/L	1.27 ± 0.87	1.28 ± 0.97	0.949
C0/C2 ratio	0.39 (0.27 - 0.55)	0.44 (0.31 - 0.67)	0.002
UA, μmol/L	295.36 ± 69.26	330.55 ± 110.30	< 0.001
Serum creatinine, μmol/L	72.34 ± 12.76	83.99 ± 26.50	< 0.001
Urinary creatinine, μmol/L	7310 (5346.75 - 10355.75)	4888(3624 - 7013)	< 0.001
ACR, mg/g	13.32 (8.99 - 19.86)	83.84 (44.76 - 277.32)	< 0.001
eGFR, ml/min/1.73 m2	80 ± 9	74 ± 14	< 0.001

SBP, systolic blood pressure; DBP, diastolic blood pressure; BMI, body mass index; ACEI, angiotensin converting enzyme inhibitor; ARB, angiotensin receptor blocker; HbA1c, glycated hemoglobin; HDL-C, high-density lipoprotein cholesterol; LDL-C, low-density lipoprotein cholesterol; C0/C2 ratio, fasting C-peptide-to-2h C-peptide ratio; UA, uric acid; ACR, albumin-to-creatinine ratio; eGFR, estimated glomerular filtration rate.

“-” means “not applicable”.

### 3.2 The association of C-peptide and UA with renal dysfunction odds

On the whole, the levels of fasting C-peptide, C0/C2 ratio and UA were significantly related with the probability of renal dysfunction in patients with T2DM in univariate logistic regression analysis using the continuous values of these three parameters. After adjustment for the potential confounders (age, gender, SBP, DBP, BMI, HbA1c level, diabetic duration, UA, fasting C-peptide and hypertension), the strength of this association remained statistically significant (**Table 2**). Every 1 SD increase of fasting C-peptide, C0/C2 ratio and UA, the odds of renal dysfunction increased by about 34%, 28% and 49%, respectively. (The SD of fasting C-peptide, C0/C2 ratio and UA were 0.30 nmol/L, 0.32 C0/C2 and 91.45 μmol/L, respectively.) However, in the multivariate analysis of these three stratified parameters (quartiles, Q1 - Q4), the participants with higher levels (Q2 - Q4) of the three indicators did not present a statistically significant differences of renal impairment odds compared with the subjects with the lowest levels (Q1) of these three parameters (p ≥ 0.05), respectively. In addition, the interaction effect between fasting C-peptide and UA for renal dysfunction odds was also performed in the logistic regression analyses. After adjustment for the potential confounders, no statistical interaction effect was found (OR = 1.09, p = 0.339), indicating that there was no multiplication interaction for the renal dysfunction odds between fasting C-peptide and UA.

**Table 2:** The associations of fasting C-peptide, C0/C2 and UA with the odds of renal dysfunction in patients with type 2 diabetes.

Variables	Univariate analyses		Multivariate analyses#	
	OR (95% CI)	p-value	OR (95% CI)	p-value
Fasting C-peptide (nmol/L)	2.18 (1.14 - 4.18)	0.018	2.64 (1.17 - 5.97)	0.019
*Continuous, per SD	1.27 (1.04 - 1.56)	0.018	1.34 (1.05 - 1.72)	0.019
Q1 (0.02 - 0.25)	1.0 (reference)	–	1.0 (reference)	–
Q2 (0.26 - 0.41)	0.71 (0.41 - 1.22)	0.216	0.75 (0.41 - 1.38)	0.352
Q3 (0.42 - 0.63)	0.48 (0.27 - 0.85)	0.011	1.09 (0.58 - 2.05)	0.784
Q4 (0.64 - 1.53)	0.63 (0.36 - 1.10)	0.106	1.55 (0.81 - 2.98)	0.191
C0/C2 ratio	2.44 (1.23 - 4.82)	0.011	2.15 (1.04 - 4.44)	0.038
**Continuous, per SD	1.34 (1.07 - 1.67)	0.011	1.28 (1.01 - 1.62)	0.038
Q1 (0.09 - 0.26)	1.0 (reference)	–	1.0 (reference)	–
Q2 (0.27 - 0.41)	1.16 (0.66 - 2.02)	0.614	0.83 (0.45 - 1.55)	0.563
Q3 (0.42 - 0.61)	1.50 (0.86 - 2.61)	0.156	0.99 (0.52 - 1.92)	0.982
Q4 (0.62 - 3.26)	2.09 (1.20 - 3.64)	0.009	1.52 (0.84 - 3.07)	0.152
UA (μmol/L)	1.004 (1.002 - 1.007)	< 0.001	1.004 (1.002 - 1.007)	0.001
***Continuous, per SD	1.50 (1.21 - 1.86)	< 0.001	1.49 (1.17 - 1.91)	0.001
Q1 (148 - 249)	1.0 (reference)	–	1.0 (reference)	–
Q2 (250 - 300)	0.65 (0.37 - 1.13)	0.127	0.68 (0.37 - 1.26)	0.223
Q3 (301 - 355)	0.83 (0.47 - 1.44)	0.502	0.79 (0.43 - 1.46)	0.453
Q4 (356 - 834)	1.80 (1.04 - 3.13)	0.037	1.59 (0.83 - 3.05)	0.161

^#^Adjusted for age, gender, SBP, DBP, BMI, HbA1c level, diabetic duration, and hypertension (yes or no), UA, fasting C-peptide; *1 SD = 0.30 nmol/L; **1 SD = 0.32 C0/C2; ***1 SD = 91.45 μmol/L; C0/C2 ratio, fasting C-peptide-to-2h C-peptide ratio; UA, uric acid; OR, odds ratio. SBP, systolic blood pressure; DBP, diastolic blood pressure; BMI, body mass index; HbA1c, glycated hemoglobin.

“-” means “not applicable”.

### 3.3 RCS analysis based on logistic regression

**Figure 1:**
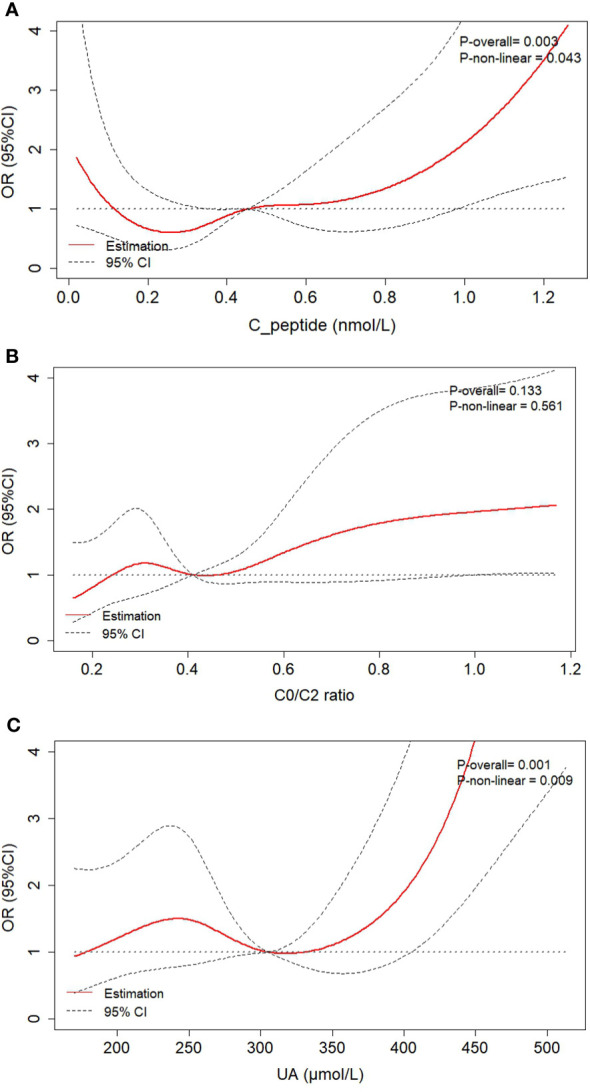
The RCS analysis based on logistic regression showed the association of renal dysfunction odds with the levels of Fasting C-peptide **(A)**, C0/C2 ratio **(B)** and UA **(C)** after adjustment for age, gender, SBP, DBP, BMI, HbA1c level, diabetic duration, and hypertension (yes or no). RCS, restricted cubic spline; C0/C2 ratio, fasting C-peptide-to-2h C-peptide ratio; UA, uric acid; SBP, systolic blood pressure; DBP, diastolic blood pressure; BMI, body mass index; HbA1c, glycated hemoglobin.

**Figure 1** showed the association of renal dysfunction odds with the levels of fasting C-peptide, C0/C2 ratio and UA after adjustment for the potential confounders. The non-linear relationships of fasting C-peptide (p_non-linear_ = 0.043) and UA (p_non-linear_ = 0.009) with the renal dysfunction odds were observed, however, there was no non-linear relationship between C0/C2 ratio and the renal dysfunction odds (p_non-linear_ = 0.561). These results indicated that although the odds of renal impairment increase with the increment of fasting C-peptide and/or UA levels in general, the relationships were not linear. Based on the RCS curves, both of the UA and fasting C-peptide variables were considered to divided into 3 categories for selecting the optimal cut-points, respectively. Finally, the optimal cut-points for UA were 276 and 409 μmol/L, and the optimal cut-points for fasting C-peptide were 0.28 and 0.56 nmol/L.

### 3.4 Threshold effects of fasting C-peptide and UA for renal dysfunction odds

The interval effects of fasting C-peptide and UA for renal dysfunction odds were evaluated using logistic regression analysis (**Table 3**). After adjustment for age, gender, SBP, DBP, BMI, HbA1c level, diabetic duration, UA and hypertension (yes or no), the odds of renal impairment in the patients with fasting C-peptide < 0.28 nmol/L and patients with fasting C-peptide > 0.56 nmol/L relatively increased by about 38% and 85%, respectively, compared with that in the patients with 0.28 ≤ fasting C-peptide ≤ 0.56 nmol/L. Compared with patients with 276 ≤ UA ≤ 409 μmol/L, patients with UA > 409 μmol/L significantly increased 5.24-folds odds (p < 0.001) of renal dysfunction, although the increase of renal dysfunction odds in the subjects with UA < 276 μmol/L had no statistical significance (OR = 1.32, p = 0.262).

**Table 3:** Threshold effect analyses of fasting C-peptide and UA on the odds of renal dysfunction in patients with type 2 diabetes.

Variables	Univariate analyses		Multivariate analyses^#^	
	OR (95% CI)	p-value	OR (95% CI)	p-value
Fasting C-peptide (nmol/L)				
0.28 - 0.56 nmol/L	1.0 (reference)	–	1.0 (reference)	–
< 0.28nmol/L	1.55 (0.95 - 2.52)	0.080	1.38 (0.81 - 2.38)	0.246
> 0.56 nmol/L	2.02 (1.26 - 3.23)	0.003	1.85 (1.10 - 3.12)	0.021
UA (μmol/L)				
276 - 409 μmol/L	1.0 (reference)	–	1.0 (reference)	–
< 276 μmol/L	1.29 (0.84 - 1.97)	0.238	1.32 (0.81 - 2.13)	0.262
> 409 μmol/L	6.19 (3.00 - 12.80)	< 0.001	6.24 (2.88 - 13.49)	< 0.001

^#^Adjusted for age, gender, SBP, DBP, BMI, HbA1c level, diabetic duration, and hypertension (yes or no), UA, fasting C-peptide; UA, uric acid; OR, odds ratio; SBP, systolic blood pressure; DBP, diastolic blood pressure; BMI, body mass index; HbA1c, glycated hemoglobin.“-” means “not applicable”.

## 4 Discussion

In the present study, we found that although fasting C-peptide, C0/C2 ratio and UA were independently associated with the odds of renal dysfunction in patients with T2DM, the probability of renal dysfunction did not linearly rise with the increase of serum fasting C-peptide and UA levels. Based on the RCS analysis, the renal impairment risk was non-linearly associated with the fasting C-peptide and UA levels after adjustment for the potential confounders, including age, gender, SBP, DBP, BMI, HbA1c level, diabetic duration, and hypertension (yes or no). Specifically, when 0.28 ≤ fasting C-peptide ≤ 0.56 nmol/L and/or when 276 ≤ UA ≤ 409 μmol/L, patients with T2DM might have the relatively lower renal dysfunction odds.

Chronic kidney disease is a very common complication of T2DM in clinical practice, its early diagnosis, intervention and treatment may have a critical impact on the delay of its progression to end-stage renal disease and the improvement of the patients prognosis (22). In recent years, the physiological function of C-peptide has been gradually discovered (11), C-peptide may block the activity of renal Na^+^-K^+^-ATPase, thus decreasing the Na^+^ reabsorption by the proximal convoluted tubule and the glomerular filtration pressure (9); the decrease of C-peptide may also promote the occurrence of microvascular complications in patients with diabetes mellitus (23). Therefore, some researchers had focused on exploring the effects of exogenous human C-peptide supplementation on the improvement of DKD (24–26). A clinical trial included twenty-one normotensive patients with microalbuminuria demonstrated that combined human C-peptide (600 nmol/24 h) and insulin administration for 3 months might improve the kidney function by decreasing the urinary albumin excretion in patients with type 1 diabetes mellitus (24). An animal model study showed the similar findings that the supplementation of exogenous C-peptide could reduce the urinary albumin excretion and inhibit the upregulation of type IV collagen *via* interaction with the TGF-beta signal in glomerular podocytes in mouses with type 1 diabetes mellitus (25). However, Samnegård B et al. conducted an animal study of 9 rats without diabetes and 22 rats with type 1 diabetes mellitus, they found that neither the albuminuria level nor the glomerular basement membrane had statistically significant improvement after the supplementation of exogenous C-peptide for 4 weeks (26). Most these previous study for C-peptide effects on DKD were conducted in patients or animals with type 1 diabetes mellitus (24–26). In addition, the C-peptide levels fluctuated greatly in different stages of T2DM (27), therefore, the role of C-peptide in renal injury in patients with T2DM is still controversial and needs more studies.

In the present study, the association of C-peptide with the renal dysfunction odds in patients with T2DM was investigated. Compared with 2h postprandial C-peptide, fasting C-peptide was independently and significantly related with the renal dysfunction prevalence. Although on the whole the odds of renal impairment raised with increase of fasting C-peptide in the multivariate regression analysis using the continuous values, a non-linear relationship was showed between the renal dysfunction odds and the different stratification of fasting C-peptide. The study of Huang Y, et al. (23) demonstrated that among the patients with T2DM, these one with 1.71 ≤ C-peptide < 2.51 ng/mL had a higher glycemic control rate, however, it did not clarify the specific relationships between the stratified C-peptide levels and the renal dysfunction odds. In this study, our findings showed that the patients with 0.28 ≤ fasting C-peptide ≤ 0.56 nmol/L had lower renal impairment chance compared with the patients with fasting C-peptide < 0.28 nmol/L or fasting C-peptide > 0.56 nmol/L. It indicated that moderate level of fasting C-peptide might be a clinically protective factor for renal impairment, on the contrary, low-level and excessive fasting C-peptide might be the risk factors for renal dysfunction in patients with T2DM. In addition, to a certain extent, the changes between fasting and postprandial C-peptide levels were correlated with the improvement of patient prognosis after intensive therapy, which reflected the reserve function of pancreatic β cell (28). Therefore, in the present study the correlation between C2/C0 ratio and renal dysfunction odds was also analyzed. Unlike fasting C-peptide, C2/C0 ratio did not show a statistically significant non-linear relationship with the odds of renal dysfunction.

A number of previous studies had shown the association between the UA levels and the kidney disease (6, 29). The mechanisms of UA induced kidney disease had been identified by several preclinical studies (29, 30). High UA levels induced and accelerated renal disease on their own without the deposition of UA crystals (29). Reducing the UA level by using xanthine oxidase inhibitor or a uricosuric agent in rats could prevent renal hypertrophy, and proteinuria (30). However, the clinical trials showed the divergent findings of the UA effects for predicting the incidence and development of chronic kidney disease in patients with diabetes (15, 16). In the present study, we found that on the whole, the higher UA level was correlated with increased incidence of renal dysfunction, with a 49% increase in renal dysfunction odds for each SD (1 SD = 91.45 μmol/L) increment independently of potential confounders. In addition, to the best of our knowledge, there are no studies that have reported the U-shape association of serum UA level with renal dysfunction odds in patients with T2DM. Our findings showed the non-linear relationship between them, indicating that in a proper range, the renal impairment incidence did not rise with the increment of UA levels. Additionally, the previous studies had indicated that fasting C-peptide was significantly associated with UA in patients with T2DM (31, 32), however, no multiplication interaction effect between these two clinical factors for the renal dysfunction odds was found in the present study.

The highlights of the present study were that we conducted a real-world study and clarified the non-linear associations of fasting C-peptide and UA with the incidence of renal impairment in patients with T2DM. However, several potential limitations of this study are worth noting. First, we did not assess *post-hoc* statistical power and the statistical power achieved for the main outcome measure in the present study. Second, this was a cross-sectional observational study, these findings could not provide strong evidences of the non-linear associations of the new-onset occurrence odds of renal dysfunction with the C-peptide and UA levels. Generally, this exploratory study highlighted and paved the way for the need to design and conduct a prospective confirmatory study in a random sample with the proper dimension that demonstrated evidence of a non-linear association of new cases of renal dysfunction with C-peptide and UA levels.

## 5 Conclusion

In conclusion, the odds of renal dysfunction was non-linearly associated with the levels of serum fasting C-peptide and UA. When 0.28 ≤ fasting C-peptide ≤ 0.56 nmol/L and/or when 276 ≤ UA ≤ 409 μmol/L, patients with T2DM might have the relatively lower renal dysfunction odds. Our results suggested that fasting C-peptide and UA might have the potential role in odds stratification of renal dysfunction, and provided laboratory evidence for prevention and treatment of renal disease in patients with T2DM and no primary kidney diseases, serious autoimmune diseases, liver diseases and/or infectious diseases.

## Data availability statement

The raw data supporting the conclusions of this article will be made available by the authors, without undue reservation.

## Ethics statement

The studies involving human participants were reviewed and approved by Ethics Committee of Affiliated Jinhua Hospital, Zhejiang University School of Medicine. The patients/participants provided their written informed consent to participate in this study.

## Author contributions

Study design: LC, YH, and HW; Statistical analysis: HW and YM; Manuscript writing: LC and HW; Data collection: LC and YH. All authors contributed to the article and approved the submitted version.
